# ‘Drawing aside the curtain’: natural childbirth on screen in 1950s Britain

**DOI:** 10.1017/S0007087417000607

**Published:** 2017-09

**Authors:** SALIM AL-GAILANI

**Affiliations:** * Department of History and Philosophy of Science, University of Cambridge, Free School Lane, Cambridge, CB2 3RH, UK. Email: ssa32@cam.ac.uk.

## Abstract

This article recovers the importance of film, and its relations to other media, in communicating the philosophies and methods of ‘natural childbirth’ in the post-war period. It focuses on an educational film made in South Africa around 1950 by controversial British physician Grantly Dick-Read, who had achieved international fame with bestselling books arguing that relaxation and education, not drugs, were the keys to freeing women from pain in childbirth. But he soon came to regard the ‘vivid’ medium of film as a more effective means of disseminating the ‘truth of [his] mission’ to audiences who might never have read his books. I reconstruct the history of a film that played a vital role in teaching Dick-Read's method to both the medical profession and the first generation of Western women to express their dissatisfaction with highly drugged, hospitalized maternity care. The article explains why advocates of natural childbirth such as Dick-Read became convinced of the value of film as a tool for recruiting supporters and discrediting rivals. Along the way, it offers insight into the British medical film industry and the challenges associated with producing, distributing and screening a depiction of birth considered unusually graphic for the time.

Graphic childbirth scenes are familiar to present-day viewers of reality television, commonplace in school sex education and ubiquitous in prenatal preparation classes.^1^ This is far from being a recent phenomenon: the screen has served as a vehicle for communicating ideas about maternity since the earliest days of cinema and television. Filmmakers have used ‘clinical’ images of the birth process to push the frontiers of cinematic expression, challenging distributors’, censors’ and viewers’ understandings of aesthetics, reality and genre. Childbirth films played a crucial role in both early twentieth-century exploitation cinema and experimental television in the 1950s, prompting considerable debate over the boundaries between education and entertainment and what counted as the proper venue and medium for instructing the public in matters of sex and reproduction.^2^ This essay seeks to further illuminate these negotiations by reconstructing the making and reception of a film that can claim particular significance in the intersecting histories of childbirth, educational cinema and television.

Within a decade of the Second World War's end, hospitals and physicians had come to define how the overwhelming majority of women in industrialized countries delivered their children. Previously places where only urban charity cases had their babies, maternity hospitals now set the parameters for the experience of childbirth for all. By the middle decades of the twentieth century, consumer demand for labour pain relief combined with institutional and social pressures to make a profusion of sedatives, analgesics and anaesthetics routine in hospitalized obstetric care. Access to pain-relieving drugs symbolized the promise of maternity hospitals to offer the ultimate in safe and modern medical practice. Yet growing numbers of women, both individually and collectively, began to express dissatisfaction with the treatment they received. Private frustrations had by the 1950s turned into a chorus of complaint about the alienating and disempowering experience of high-technology obstetrics.^3^ Many among this first generation of women to voice their disillusionment with medicalized maternity care sought an alternative way of birth in a suite of approaches based on the rejection or minimal use of pharmacological pain relief, described generically as ‘natural childbirth’.^4^

In the post-war decades, natural childbirth was virtually synonymous with the British physician Grantly Dick-Read (1890–1959) and his French rival Fernand Lamaze (1891–1957). These were competing systems with distinct origins; Dick-Read, Lamaze and their followers fought bitterly to differentiate the two approaches. Nevertheless, these controversial reformers shared an antipathy toward the routine use of pharmacological pain relief in normal birth, promoting measured breathing techniques and preparatory routines that diverged only in small ways. Although Lamaze's method achieved greater long-term success, both physicians attracted a huge following in the 1950s and beyond, bringing debate about obstetric pain relief and the management of labour to the international stage. Dick-Read, Lamaze and their promoters undoubtedly tapped into a genuine desire for change in maternity care among childbearing couples in many countries, but historians are only beginning to attend to the processes of communication that made the ideal of unanaesthetized birth so resonant.^5^ Bestselling books, newspaper columns, magazine articles, public lectures, radio and television broadcasts, educational films and theatrical motion pictures were not merely methods of transmission or vectors of individual fame but, taken together, were constitutive of natural childbirth as practice and international phenomenon.^6^ Reconstructing these strategies for engaging consumers and the historical contingencies that made them possible is no less important than understanding natural childbirth as an ‘ideology’ or discourse.^7^

Both Dick-Read and Lamaze, as well as generations of their followers, regarded film as a powerful vehicle not only for persuading the world of the efficacy of their approaches, but also for instructing expectant mothers and their partners. Central to both systems was an emphasis on psychological preparation for childbirth through prolonged prenatal education, which Dick-Read and Lamaze considered essential to easing women's fears and managing labour pain without resorting to anaesthetics. In the 1950s, both obstetricians produced films designed for the purpose of preparing pregnant women for labour. These films, and others inspired by them, were key components of the prenatal education classes organized by medical and lay groups who took up their cause. Films have remained integral to childbirth preparation ever since.^8^

This essay concentrates on one of the prototypes for the childbirth preparation film. Made in South Africa and Britain at the height of the post-war educational-film boom, Dick-Read's self-funded *Childbirth without Fear* played a crucial role in communicating his methods ‘practically’ and ‘visually’ to nurses, midwives, sympathetic doctors and expectant mothers during the 1950s and early 1960s.^9^ Like many medical films, *Childbirth without Fear* circulated beyond the clinical domain. Lay members of the British Natural Childbirth Association (today the Natural Childbirth Trust or NCT), among the first activist groups to campaign for maternity service reform, demanded the right to view the film at their meetings from 1956. In 1957, a short clip from the film reached a mass audience as part of a debate on natural childbirth on BBC television's flagship current-affairs programme *Panorama*. The first footage of an ‘actual birth’ to be broadcast on British television, the feature made headlines across the world. *Childbirth without Fear* therefore provides a lens through which to explore how communication about sex and reproduction was bound up in intensified exchange between different media, and between medicine and the media in the middle decades of the twentieth century. The dynamics between print, educational film and television played an important part in reshaping expectations about the experience and visibility of birth in post-war British culture.

## Grantly Dick-Read and the politics of labour pain management

The term ‘natural childbirth’ derives from the title of a short 1933 treatise by Grantly Dick-Read.^10^ In this and several other books and articles published over the next quarter-century, the British-born physician outlined an alternative to the anaesthetized, medically controlled way of birth common among Western women of privilege, based on the premise that fear lay at the root of pain in labour.^11^ For Dick-Read, whether or not a mother experienced pain in labour depended not on some property inherent to the physiology of parturition but on cultural attitudes to childbirth. Culture and civilization, he argued, had conspired to distort Western women's capacity for pain-free birth. Through education and relaxation women could overcome what he termed the ‘Fear–Tension–Pain’ cycle and labour in comfort without resorting to medical intervention. Preparation for labour meant providing pregnant women with detailed instruction, from their physician, midwife or qualified childbirth educator, on the physiology of pregnancy and birth, nutrition, exercise, hygiene and infant care. In the months prior to birth, the expectant mother was to practice relaxation through prescribed daily exercises, through which she could learn to discipline her emotions, embrace the prospect of labour with joy and see her active role in the birth process as critical to a successful delivery. Dick-Read promised women a fulfilling personal experience, a more intimate bond with their babies, and happy, healthier children.

Dick-Read's approach, or the ‘Read method’, as it was described in the popular literature of the 1940s and 1950s, had precedents in the work of earlier health reformers and obstetricians who had proposed holistic alternatives to the prevailing model of highly drugged, interventionist childbirth.^12^ But his books, translated into more than a dozen languages, brought the concept of unanaesthetized labour to an international readership of unprecedented scale. One reviewer estimated in 1950 that ‘no [other] medical author [had] been so extensively read by laymen’.^13^ Framed by eugenic concerns about low birth rates among the ‘over-civilised’ middle and upper classes and fired by an evangelical faith in the spiritual significance of motherhood, Dick-Read's writings resonated in a post-war climate that venerated family life, and women's identities as consumers and homemakers. His emphasis on the role of husbands – practically unseen in delivery rooms before the 1950s – in providing support to their wives during pregnancy and labour chimed with the mid-century ideal of ‘companionate marriage’, appeals for more satisfying emotional relationships between spouses, and men's responsibility to engage in more active parenting roles.^14^ If the war eviscerated old assumptions about gender relations, it also produced a hunger to learn about how personal security could be established and maintained through the institution of the family, and made an emblem of national health and a bulwark against communism. Dick-Read was one of a host of experts on mothering and family life brought to prominence during the post-war years; listening to such authorities was encouraged as one of women's central tasks in creating a good home.^15^

Dick-Read earned an enthusiastic following, especially in Britain and the United States, inspiring millions of prospective mothers to seek a birth experience that was atypical for urban, Western women of privilege. Dick-Read's ideas were also hugely controversial, provoking reprobation from medical colleagues horrified by his critique of techniques and technologies they believed had made pregnancy and childbirth safer. Although he enjoyed some support among sympathetic obstetricians, critics reckoned that Dick-Read's claims were based at best on anecdotal evidence devoid of the scientific trappings expected of medical research, and at worst on ‘assumption, factual error and wishful thinking’.^16^ Moreover, medical practitioners at this time were strongly discouraged from self-promotion, and many colleagues viewed Dick-Read's active cultivation of the media and direct engagement with lay audiences as a flagrant violation of the professional ethic of anonymity.^17^ Considered an anachronism by the British medical establishment for his eccentric emphasis on spiritual motherhood and anti-technological rhetoric, Dick-Read failed to gain the official recognition he believed he deserved. Disillusioned with the lack of support from the Royal College of Obstetricians and Gynaecologists and pessimistic about his prospects within the post-war National Health Service, he resolved in 1948 to leave Britain to pursue a new venture in South Africa.^18^

Working at a small Catholic maternity hospital in Johannesburg, Dick-Read first learned of a new school of obstetricians in France promoting psychological methods of pain relief imported from the Soviet Union. Grounded theoretically in physiologist Ivan Pavlov's concept of conditional response and developed independently of Dick-Read, ‘psychoprophylaxis’ offered Soviet obstetricians an attractive alternative to pharmacological anaesthetics amidst medical personnel and pharmaceutical supply shortages in the wartime and post-war years in which maternal care was a low priority for the central authorities. Convinced by the apparent efficacy of the method, Fernand Lamaze promoted psychoprophylaxis in France as ‘l'accouchement sans douleur’ from his maternity ward at Les Bluets, the Paris Metallurgists’ Polyclinic. What became known as the ‘Lamaze method’ achieved rapid success, historian Paula Michaels has argued, nurtured by a long-standing medical and state concern with the declining birth rate, and endorsed by the French Communist Party as an achievement of Soviet science. Networks of communist organizations, Michaels shows, were instrumental in sparking public interest in psychoprophylaxis and making Les Bluets the national and international centre of the technique in the 1950s.^19^

By 1952, Dick-Read had launched a sustained attack on Lamaze and his school. He resisted his French counterpart's attempts to assimilate natural childbirth into the growing psychoprophylactic movement and, for obvious reasons, fended off suggestions that Soviet obstetricians possessed greater claims to priority or recognition for psychological approaches to obstetric pain relief. Dick-Read not only accused Lamaze and his Soviet precursors of plagiarism, but also condemned psychoprophylaxis as unnatural and anathema to the ideal of spiritual motherhood. Dick-Read's vocal anti-communism framed an increasingly strident, but largely ineffectual, struggle against psychoprophylaxis and the ‘atheistic and materialistic’ ideology underpinning it.^20^ To anyone but the most invested in one method or the other, the approaches appeared virtually indistinguishable and were frequently confused. But to Dick-Read, and increasingly also promoters of psychoprophylaxis, subtle differences were all-important.^21^

Lamaze and his school were quick to recognize the potential of a ‘cinematographic apprenticeship to painless childbirth’, making extensive use of film to instruct both lay and medical audiences in the psychoprophylactic technique from at least 1953.^22^ The growing importance of audiovisual tools in the promotion of psychoprophylaxis in the 1950s helps to explain why Dick-Read viewed film as a crucial weapon in his battle to insulate natural childbirth from what he dismissed as Soviet ‘propaganda’.^23^

## *Childbirth without Fear* between print and film

Dick-Read's relocation to Johannesburg marked the start of arguably the most productive years of his career. This included preparing several new editions and overseeing translations of his bestselling book *Childbirth without Fear*, first published by Heinemann under the title *The Revelation of Childbirth* in 1942. By 1954 he had given up clinical practice and had returned to Britain effectively as a full-time author, with his entire income from book royalties and lecture fees.^24^ Before leaving South Africa, however, Dick-Read fulfilled a long-standing ambition to film a series of mothers giving birth ‘naturally’. This section outlines Dick-Read's intentions for the film and the relations between print and moving image.

Dick-Read was first drawn to the medium in the mid-1940s, seeking advice from J. Arthur Rank, Britain's leading film magnate and a casual acquaintance of the Read family.^25^ Dick-Read's success as an author also attracted several speculative offers from smaller production companies keen to collaborate in disseminating the obstetrician's ‘revolutionary idea’ to the widest possible audience.^26^ Weakly developed compared to that of the United States, the British educational and medical film industry took off during the Second World War with support from the British Council, the British Film Institute and the new Scientific Film Association. By war's end, government film services, voluntary health associations, medical-equipment companies and industrial sponsors worked to build awareness of the medium among researchers and educators, and streamline systems of production and distribution.^27^ Despite the growth of the industry around this time, Dick-Read's efforts in film were typical of an early post-war scene still largely dominated by ‘isolated enthusiasts rather than any concerted professional initiative’.^28^ Many medical teachers and health workers viewed the significant expense and effort involved in producing such films as prohibitive even as they recognized their potential.

Cost was certainly a factor for Dick-Read, initially discouraged by what seemed to him exorbitant prices of materials, recording equipment and production assistance in Britain. All of these could be procured more affordably in Johannesburg. Meanwhile, the suburban Marymount maternity hospital, far from the scrutiny of hostile colleagues and where Dick-Read enjoyed the support of the Catholic sisters who ran it, offered a congenial environment to experiment with film that he could not have expected at home. In July 1953, having already resolved to return to Britain, he arranged for a local photographer to assist with filming the last four of his six hundred cases at the Marymount. These four women – white, affluent and fully committed to natural childbirth – agreed to take part on the condition that the film would not be screened in South Africa.^29^ Dick-Read left Johannesburg soon after, in possession of the 16 mm colour film reel he believed would secure recognition for his method. Back in Britain, he reconnected with Rank, whose production company Gaumont-British Instructional helped turn the raw footage of the four unanaesthetized deliveries into a ‘first-rate educational film’.^30^

By around 1950, there was already a substantial stock of obstetric films available to professional audiences, mostly demonstrating surgical or anaesthetic techniques.^31^ But British obstetricians were often sceptical about the pedagogical value of such films, which many complained became outdated on points of practice as quickly as they were made.^32^ A far better use of film, *The Lancet* suggested in 1951, was to make unadorned records of clinical cases unencumbered by therapeutic claims.^33^ Even though his close-up, graphic record of childbirth most resembled these cinematic case histories, Dick-Read wanted his footage to be ‘exhibited universally’.^34^ Medical professionals and film censors generally considered the step-by-step portrayal of the actual birth of a baby inappropriate for lay audiences, but there is evidence of shifting attitudes by the 1950s. The controversial American exploitation film *Birth of a Baby* (1938), which included intercut documentary footage of an actual birth, was initially rejected by British censors but received broad medical and journalistic approval when rereleased in 1947.^35^ Even so, censors generally deemed ‘clinical shots of this nature … [unsuitable] for inclusion in any film shown to the general cinema public’.^36^

**Figure 1.**
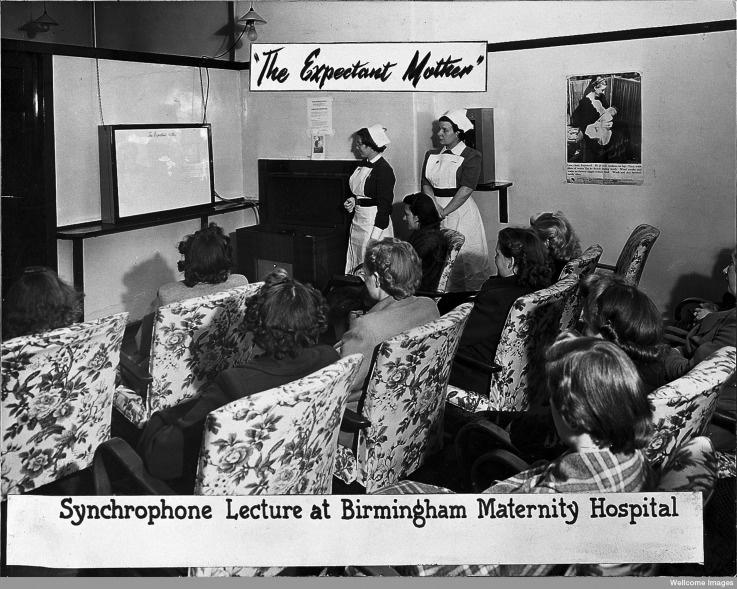
Photograph of a ‘synchrophone lecture’ for expectant mothers at the Birmingham Maternity Hospital, taken around 1945. The synchrophone was a projector widely used to exhibit educational films with sound in the 1930s and 1940s. Wellcome Library, London: National Birthday Trust Fund Archive, SA/NBTF G31/7/5.

Within these constraints, the viewership foremost in Dick-Read's mind was a ‘non-theatrical’ audience of pregnant women attending antenatal, baby welfare and mothercraft clinics run by voluntary and local-authority health associations. Antenatal services had expanded rapidly during the war, with around two thousand clinics offering some form of supervision to nearly all expectant mothers in Britain by 1944.^37^ The clinics provided a context for prenatal health instruction, with organizers keen to experiment with new audiovisual tools to enliven their ‘pep talks’ and satisfy women's desire ‘to know what actually happens at birth’ (Figure 1).^38^ By targeting this audience, Dick-Read most closely aligned his ambitions for the film with a growing network of professionals and voluntary groups involved in health communication, many with long-standing interests in exploiting the educational potential of cinema.^39^ John Burton, medical director of the local-authority-funded Central Council for Health Education, was a keen advocate for the role of film in public health, writing frequently on the potential of cinema to exert a ‘mass influence’ on the ‘young family’ in particular.^40^

But this influence was for health educators a double-edged sword. Was film, Burton asked in 1953, ‘a drug or an informative experience? An incentive to sadistic crime or a harmless escape from reality?’^41^ These reservations came out of long-standing anxieties about the dangers of motion picture spectatorship and in particular the suggestive power of the moving image to implant socially undesirable ideas in the minds of viewing audiences.^42^ Obstetricians had for decades expressed concern about the ‘emotional stimulus’ of film in heightening ‘nervous tension’ in expectant mothers. Dick-Read himself railed against the pernicious effects of mainstream depictions of childbearing on prospective parents, and especially young women. ‘There seems to be a demand by those who produce such pictures to publish to the world the sufferings of women in labour’, he complained, and these could only serve to heighten women's fear of confinement.^43^ Pronatalist fears that graphic images of childbirth, particularly on screen, would make motherhood ‘unattractive’ proliferated throughout the mid-twentieth century and continued to frame conservative critiques of sex education long into the ‘permissive’ era.^44^

Dick-Read wanted more positive representations of childbirth to act as counterweights to the dominant cinematic tropes. The presumed capacity of film to directly address viewers’ emotions with unique persuasive power had long underpinned arguments about the risks and benefits of cinema, both in the context of medical education and in mass communication.^45^ This emphasis on the affective qualities of the moving image informed Dick-Read's conviction that the medium was especially well suited to communicate a method concerned first and foremost with the ‘the care of the emotional states of the woman’ in pregnancy and labour. Given that cinema had such ‘considerable influence on the minds of the people’, film promised to reach wider audiences, even more effectively, than the written word.^46^

In adopting film, Dick-Read also sought to address criticism from other health professionals that his methods were unscientific and unproven. In his popular books, he insisted that he wrote ‘not in academic dogma, but rather [as] one who records clinical observations’. By including case reports in an appendix to *Childbirth without Fear*, Dick-Read treated these ‘accurate scientific observations’ both as a measure of efficacy and as a tool of persuasion. He described using a sound recorder as a useful device for making ‘spontaneous’ notes of his cases.^47^ The camera was a tool for reporting the various phenomena of labour in a manner that was still more immediate than was possible in print.^48^ ‘Here was incontrovertible proof of the practice of [natural childbirth] – vivid, visual, documentary evidence for medical men, students and nurses everywhere’.^49^ Dick-Read's surviving correspondence with editors at Gaumont-British Instructional underscores his unwavering belief in the film as an objective, unmediated record of natural childbirth. Delighted with the final result, he gratefully wrote to Rank that ‘this picture will be one of the strongest influences in establishing the truth of my mission to women’.^50^ The film was ready to screen in summer 1954, distributed by another subsidiary of the Rank Organization, Gaumont-British Equipments, under the title *Childbirth without Fear*.^51^

Dick-Read's investment in the moving image can be understood as part of a more adventurous and experimental publication strategy that coincided with his return to Britain from South Africa. In the 1940s, Dick-Read was known primarily through his books. Giving up clinical practice meant added pressure, but also greater freedom, to pursue alternative sources of income and build his public profile. Dick-Read continued to work on new editions of his books, but in conjunction with his writing he sought to exploit new media platforms. In summer 1954, the British tabloid *News of the World* serialized Dick-Read's account of the ‘safari’ he made by caravan to record childbirth practices among tribal women of central Africa. Dick-Read wrote enthusiastically about what he termed ‘normal childbirth in its natural environment’, the wisdom of the ‘primitive African woman’ and the ‘fearless desire for children’ that allowed her to sail through pregnancy and labour with comfort. Based on long-standing myths about the ‘primitive woman’ and her ability to give birth with ease, the travel narrative would not only form the substance of his last book, *No Time for Fear*, but also shape the presentation of the film and wider promotion of the Read method in the later 1950s.^52^ The *News of the World* articles signalled a more concerted effort to reach broader audiences and document the ‘truth’ of natural childbirth in novel ways: through, for instance, women's magazines, radio and television appearances, public lectures, sound recordings, photographs and filmstrips. Each of these platforms conveyed various meanings of ‘natural childbirth’ to a wide spectrum of constituencies. What can we learn about these meanings from his ventures in film?

## Witnessing ‘mother-love’

Burdened with the ‘serious personal expense’ of producing the film, Dick-Read was heavily invested in its financial success.^53^ This meant designing and promoting the picture for and to audiences who might purchase or hire prints. Negotiations between Dick-Read, distributors Gaumont-British Equipments, and the various parties interested in the film illuminate the practical challenges involved with bringing *Childbirth without Fear* to screen. What did viewers of Dick-Read's film see, and how did he attempt to shape the response?

First screened at a medical congress in Geneva in summer 1954, *Childbirth without Fear* is a full-colour, twenty-minute-long ‘documentary record’ of the last four cases of Dick-Read's Johannesburg practice. The women are shown giving birth without anaesthetics or instruments, under Dick-Read's supervision. While the strength and dignity of the labouring women, and the supportive presence of their husbands, are emphasized, so too is Dick-Read's calm authority, professional judgement and control of the delivery room.^54^ Nothing is explained of the preparatory instruction or relaxation exercises supposedly constitutive of the Read method; the film concentrates instead on the labour and delivery sequences, providing graphic footage of the emergence of the baby and the delivery of the placenta while keeping the mothers’ faces and reactions in view. The film opens with a short account of natural childbirth by Dick-Read, who also provides a recorded voice-over describing the birth process and guiding the viewer to look for signs that the labour is progressing satisfactorily.^55^

With moving pictures of childbirth deemed too ‘frightening for young expectant mothers’, the distributors were cautious about ‘indiscriminately’ exhibiting what they considered a straightforwardly ‘medical film’.^56^ *Childbirth without Fear* was ‘suitable only for specialised audiences’ and ‘rather dangerous’ for lay viewers ‘unless it were introduced by a suitable person’ to ‘prepare’ viewers for the graphic sequences.^57^ Staff at Gaumont-British struggled to persuade Dick-Read, who naively assumed that the picture was of ‘educational value to a very large proportion of the population of this country’, to lower his expectations since audiences for instructional films were so unpredictable. An earlier series on ‘human reproduction’ intended for schoolchildren, for instance, had been a commercial flop.^58^ For his part, Dick-Read grew increasingly frustrated with a company he thought had not grasped the film's broad appeal and, through lacklustre publicity, had failed to arouse the demand he believed it merited. In 1956, he went as far as to accuse a Gaumont-British executive of maligning the film to potential customers.^59^

Regardless of the private views of either Gaumont-British or their overseas agents, and however committed they were to marketing *Childbirth without Fear*, Dick-Read assumed the weight of the responsibility for publicizing the film. As his critics often pointed out, the popularity of natural childbirth depended so much on Dick-Read's communicative gifts, the intimacy he developed with women persuaded by his approach, and the devotion he inspired among lay supporters.^60^ During his lifetime, natural childbirth was rarely dissociated from Dick-Read the personality; similarly, his visual and verbal presence both on screen and in theatres shaped how the film was seen and heard.^61^ The picture did not speak for itself, but was only part of a wider performance.

Between 1955 and 1959, Dick-Read presented *Childbirth without Fear* at numerous private screenings in cinemas on Wardour Street, the centre of the British film industry, and subsequently on a lecture tour to various British hospitals, international professional congresses, health education summer schools, midwives’ meetings and women's clubs. As health communication experts around this time often stressed, the potential of film depended less on the intrinsic qualities of the medium than on the context and manner in which it was exhibited. John Burton of the Central Council for Health Education, by the mid-1950s a key supporter of Dick-Read and natural childbirth, insisted that films should not be regarded as ‘self-sufficient media’, exhibited to passive audiences who ‘come, see and are conquered’. On the contrary, he considered moving pictures effective in health education only when used selectively to explain an argument or as a basis for discussion, and integrated with other ‘equipment for learning’.^62^ Whether lay supporters or medical critics, audiences viewed *Childbirth without Fear* in conjunction with other presentation devices as preludes to discussion: typically a lecture by Dick-Read on the principles of natural childbirth and a synopsis of antenatal instruction by his wife, Jessica, often using other visual aids and living models to demonstrate breathing and relaxation exercises.^63^

The introductory texts, scripts and commentaries that Dick-Read prepared for the screenings give a sense of his presentation strategy. First, he would make great play of the alleged realism of the picture. ‘The photography was by men who had no previous experience in the field’, taken in a ‘labour ward in constant use and … unaltered’ for filming rather than under ‘perfect academic conditions’.^64^ The women were not ‘specially selected’ but ‘typical’ cases. The film was ‘no studio portrait’ but ‘simple, honest and impromptu photographs taken with the object of demonstrating what goes on day after day with 96 per cent of women trained to have their babies naturally’.^65^ Second, Dick-Read would relate the obstetric histories of each of the women, contrasting complex prior deliveries with the comparatively easy, relaxed and unanaesthetized birth experiences recorded on film.^66^ Third, he used the content of the film to highlight factors contributing to the birthing women's emotional well-being: the actions and comportment of the birth attendants, the mother's labour position and especially the ‘part the husband can play during his wife's confinement’. Fourth, he would outline the disadvantages of pharmacological pain relief and note the ‘obvious advantages [of natural childbirth] shown in the film’, the ‘emotional reward and physical wellbeing of women’ able to ‘watch their babies being born and hear the first cry’. Most striking to audiences at the screenings were Dick-Read's attempts to draw viewers’ attention to each mother's ‘actions, movements and expressions’. He invited his audiences to read ‘the emotional states through which she is obviously passing’, building up to what Dick-Read termed the ‘incredulous happiness’ of motherhood.^67^

Together with the apparent absence of surgical interference or anaesthetics, these cinematic records of his patients’ emotions were for Dick-Read crucial evidence of what he termed the ‘ecstasy of accomplishment’ of natural childbirth. ‘If any scientist has doubts about the theory upon which these procedures are based’, he proclaimed, ‘let these routine results occupy his attention’. By framing the film in this way, Dick-Read aimed to buttress a larger psychological argument about ‘the need for mother-love’. In step with the post-war explosion of scientific and popular writing on the importance of human emotions and their biological underpinnings in general, and mother–child relationships in particular, he claimed that only through natural childbirth could women fully bond with their babies and appreciate ‘the physical, spiritual and emotional achievement’ of motherhood. Contemporaries such as the psychoanalyst Donald Winnicott, for instance, in a series of influential BBC radio broadcasts, set out the importance of healthy mother–child relationships to the proper functioning of democracy and as a check against the disintegrating, destabilizing forces of the modern world.^68^

In a similar vein, Dick-Read used his film to claim natural childbirth as the foundation of ‘a true mother–child relationship’. His exemplars were ‘primitive tribes of Central Africa and Belgian Congo’ who, ‘guided only by the natural laws of reproduction and survival’, approached childbirth not with fear and discomfort, but with ‘confident anticipation of motherhood’. Psychologically healing and emotionally rewarding, fearless childbirth meant reasserting ‘human love’ over the mechanical, artificial trappings of modern civilization, and restoring the ‘natural’ needs of women, men and children. Not simply visual evidence of ‘spiritual motherhood’, *Childbirth without Fear* was intended to evoke similar feelings in those who viewed it; the process of witnessing itself had the capacity to be psychologically transformative. Through these four brief vignettes, the film promised to ‘draw aside the curtain with which culture has hidden both reality and truth’.^69^

## ‘The most intimate scenes ever televised’

Dick-Read worked hard to promote the film, hastily arranging previews for visiting medical practitioners, cajoling potential allies into attending screenings, and lobbying journalists. He faced two major obstacles: continued medical scepticism about his methods, and broader taboos about frank public discussion of childbirth, especially over the display of ‘clinical’ images of birth scenes. Dick-Read's complaints that medical opponents in various countries were ‘manoeuvring’ to prevent *Childbirth without Fear* being shown may have been exaggerated.^70^ Even so, the film undoubtedly failed to gain significant medical endorsement in the two critical years after its release. The exasperated distributors reported that the ‘prevailing reaction’ among professional audiences was that the picture ‘illustrates the conclusions, but does not in fact demonstrate the process or method by which the results are obtained’.^71^ Reviews in the medical press, meanwhile, cast doubt on the instructional value of the film given that Dick-Read's methods remained so controversial.^72^

Dick-Read far more successfully attracted support from the lay press, including newspapers such as *The Guardian* and the tabloid *Daily Sketch*, but more especially parenting magazines such as *Nursery World* and *Parents*.^73^ These journals provided a key forum for the discussion of natural childbirth in the mid-1950s and also played a critical role in creating demand to view the picture. Hailing the film as evidence that ‘all women can benefit from Dr. Dick Read's great teaching and have their babies in the natural way’, an editorial in *Parents* enthused that ‘all expectant mothers should go out of their way to see [it] in order to gain confidence in their own coming delivery’. The editorial called on readers to press local hospitals and antenatal clinics to get hold of copies and encourage other mothers to ‘go and see it … I have had three children and I have read a lot about childbirth, but it was not until I saw this film that I *really* knew what happened!’^74^

One of these women was Prunella Briance, a young mother living in London, who had been motivated to attempt a natural delivery having read Dick-Read's books. Feeling deeply that conventional obstetric care was to blame for the stillbirth of her baby, Briance resolved to establish an organization dedicated to promoting the Read method. Founded in May 1956, the Natural Childbirth Association campaigned for better understanding of the Read method among maternity staff, and more sympathetic treatment of women who wished to use it. A second objective was to establish a lay teaching network of voluntary antenatal clinics, run by women with experience in natural childbirth, to instruct prospective mothers in the Read method. These clinics would show the film ‘to all women wishing to see it’ and, with other audiovisual aids, ‘help mothers understand exactly what was expected of them during labour’.^75^ However, the British Council refused the association use of its cinema, its medical viewing panel having considered the film unsuitable for lay audiences.^76^

Controversy only increased when, on 4 February 1957, BBC television announced that it would broadcast a clip from the film on its flagship news magazine *Panorama* as part of a discussion on natural childbirth. That evening viewers would see, for the first time on British television, ‘the actual moment of a baby's arrival … the baby's head emerge – and then the whole child leaving the mother's body’.^77^ Issues around motherhood and family life had figured prominently on BBC television at a time when programmes for women on the new platform had assumed a particular priority within a broader effort to establish a culture of television viewing.^78^ By the mid-1950s, such innovative series as *Family Affairs* had made discussions of pregnancy and birth a staple of women's television programming, typically broadcast in the early afternoon.^79^ If the decision to devote a segment to natural childbirth on *Panorama*, then watched by one in four adults in the UK, reflected how newsworthy the matter had now become, it also gave the BBC licence to broadcast more graphic maternity sequences than were considered suitable for daytime television. Only weeks before, commenting on a new series of *Family Affairs* made in cooperation with a major London hospital, the editor of women's television programming had asserted categorically that footage of the ‘actual moment of delivery’ was ‘not suitable for public showing’.^80^ But the broadcast came in the context of an emerging ratings war between the BBC and an increasingly dominant ITV, launched in September 1955, but by 1957 claiming an 73 per cent share of the British television audience.^81^

The *Panorama* broadcast proved a ratings winner for the BBC, attracting by some distance the biggest audience on either channel that week. Soon after 9.00 p.m., an estimated eleven and a quarter million British television viewers watched a short extract from *Childbirth without Fear*, described by one newspaper as ‘the most crowded forty seconds television has ever offered’.^82^ The clip followed a studio debate chaired by *Panorama* presenter Richard Dimbleby, who was associated with the BBC's most significant political and social programming. Dick-Read and Jean Cormack, the treasurer of the new Natural Childbirth Association, clashed angrily with two critics over the film's contents, whether women stood to benefit from his techniques, and the ‘prejudice’ of the medical profession.^83^ The programme drew significant press comment, with many newspapers soliciting from the viewing public reactions to ‘the most intimate scenes ever televised’.^84^ The BBC received much criticism, with the strongest attack from the *Daily Sketch*, perversely one of the few British newspapers to have reported on the film when it was screened to the press a year previously. The tabloid's medical correspondent, Robert Shields, had enthused then that ‘every mother should see this film’. But with headlines condemning the ‘revolting’, ‘farmyard level’, ‘horror comic’ broadcast, the *Sketch* now judged the *Panorama* producers guilty of the ‘worst display of taste ever’.^85^

Although other newspapers joined the *Sketch* in charging the BBC with ‘squalid’ sensationalism and the irresponsible use of the new medium of television, dominating press commentary in the days after the broadcast was the absence of any significant public outcry.^86^ Newspapers widely quoted BBC figures as claiming that, despite bracing themselves for a ‘flood of telephone calls’, there were only a handful of complaints.^87^ Many viewers interviewed in newspapers’ ‘snap polls’ wholeheartedly approved of the decision to broadcast the clip, praising its educational value, with several expressing a wish to see the complete film. One mother from Leeds quoted in the *Yorkshire Post* was typical of proponents in claiming that she had watched the programme with her two young children: ‘far better that they should see something like that’, she reasoned, ‘than hear hole-and-corner stories about childbirth’. ‘By all means’, an ‘Edinburgh housewife’ agreed, ‘let the young mother and father-to-be and all sensibly educated children know about birth fully, so that foolish and unfounded fears surrounding it will for ever vanish’.^88^ A major discussion point was the impact of the footage on men; a haulage contractor from Manchester was quoted as saying he thought ‘all men should see what their womenfolk have to go through in childbirth’, while a member of the Family Planning Association asserted that such footage might teach husbands not to ‘take too much for granted’ and perhaps even ‘evoke more sympathy and communal understanding’ for their wives.^89^

In the absence of any detailed contemporary analysis of audience reception, this coverage offers a valuable, albeit selective, glimpse of how this televised fragment of Dick-Read's film was received by viewers. For all the furore over the BBC's sensationalism, this ‘televisual occasion’ and the responses it generated were, perhaps, symptoms of a broader shift in British sexual culture by the 1950s.^90^ The apparent enthusiasm for graphic images of childbirth echoed the broadly positive reactions of audiences to such films as *Birth of a Baby* noted by journalists and investigators working for social research organization Mass-Observation almost two decades earlier.^91^ The rapid growth of television and the wider diversification of the media combined with the 1950s consumer boom could only accelerate changes to an environment in which matters of sex were discussed more openly and more frequently.^92^ As one journalist reflected on the *Panorama* broadcast, ‘gradually, through the medium of television, the eyes of the public are being made to see more clearly’, creating conditions in which graphic images of childbirth might be ‘accepted by all ages at a purely clinical level without either sniggers or shouts of indignation’.^93^ Such images certainly became more commonplace: in July 1957 ITV televised a five-minute film of a Caesarean birth and the BBC would devote several more television programmes to the subject in the early 1960s.^94^ Meanwhile, film censors relaxed their opposition to childbirth sequences: in 1958 the British Board of Film Censors passed with an X certificate a picture rejected five years earlier, provided publicity offered adequate warnings of the ‘clinical’ content and there were no stills of the actual birth scenes shown outside cinemas.^95^

**Figure 2.**
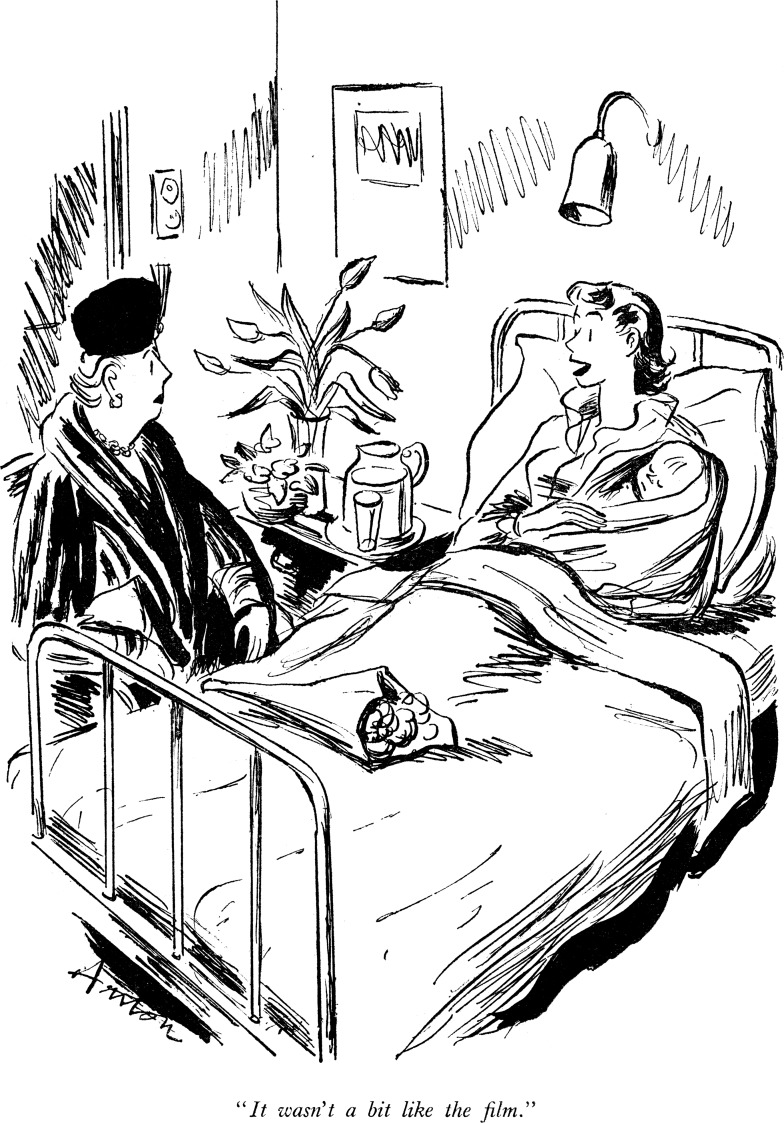
*Punch*’s satirical take on the film, published 13 February 1957. The cartoon captures a strand of criticism often levelled at Dick-Read, including on the *Panorama* debate, that he was leading women ‘up the garden path’ with ‘wild claims’ about the efficacy of his methods. Reproduced with permission of Punch Ltd, at www.punch.co.uk.

The *Panorama* broadcast had the more immediate consequence of drawing unprecedented attention to Dick-Read's film and natural childbirth. For critics, the clip only confirmed that Dick-Read's claims were ‘incapable of substantiation’ (Figure 2).^96^ But for those young mothers and nurses already sympathetic to his principles, the ‘outburst’ over the BBC broadcast was symbolic of a maternity service unresponsive to women's perceived needs and the film a compelling vision of an alternative way of birth. ‘I saw Dr. Dick-Read's film on the TV’, one enthusiastic mother wrote to *Parents*, ‘and was so excited my husband thought I was having a fit’. For those who joined the Natural Childbirth Association, the film was a tool for showing prospective mothers ‘how to have a baby the natural way’ and for improving the seeming communication gap between childbearing women and the medical profession. Prevented from showing the film at the British Council, the association hired a Wardour Street theatre for monthly screenings from May 1957. A year later, parenting magazines were still encouraging women outside London to press local maternity centres to arrange viewings.^97^

Quickly integrated into the educational apparatus of the association, *Childbirth without Fear*, then, also played a crucial role in mobilizing this first wave of maternity activists and defining their goals. For followers of the Read method, such images were necessary to show prospective mothers ‘how to have a baby the natural way’ in a context where medical attendants were typically unsupportive and women ill-informed about the process of birth.^98^ The experience of viewing the film would encourage activists to produce and make public visual records of their own labours.^99^ If the concept of childbirth as private, connected with sex and subject to similar taboos, acted as a ‘powerful sanction against public–political discussion’, securing access to and visibility for the film was part of a broader strategy to provide childbearing women with resources to articulate their demands.^100^

## Conclusion

By the time of Dick-Read's death in 1959, natural childbirth was rapidly losing ground to the psychoprophylactic technique associated with Fernand Lamaze. By the late 1950s, film had become an instrument in the wider struggle between the two approaches. Dick-Read's denunciations of films promoting psychoprophylaxis as ‘medical and political propaganda’ found a sympathetic audience in West Germany, where a particularly strident and pervasive anti-communism structured discussions around motherhood and the family.^101^ In 1957, a Frankfurt-based production company acquired the rights to exhibit *Childbirth without Fear*, hoping to combat pictures promoting the French technique then being screened in the ‘Russian zone’.^102^ But Dick-Read's hostility to psychoprophylaxis and increasingly desperate attempts to frame childbirth as a front in the Cold War proved futile, ultimately alienating many of those who had initially supported his work. By 1960, the Natural Childbirth Association (now renamed the National Childbirth Trust) had shifted its allegiance to Lamaze and fully committed to psychoprophylaxis.^103^

Nevertheless, advocates of the Read method continued to promote *Childbirth without Fear* as a teaching aid well into the 1960s.^104^ Although far from the commercial success that Dick-Read hoped it would be, his ‘documentary record’ helped secure a role for film as a tool for educating and empowering a new generation of women no longer content to accept the prevailing model of medicalized obstetric care. The expectant mother had been a target for public-health films long prior to the 1950s. Such films had promoted, and would continue to promote, awareness of new services, including hospitalized maternity care. *Childbirth without Fear* was unusual in that it was intentionally disruptive of medical orthodoxy and blurred the traditional distinction between films aimed at professional audiences and those for laypeople. For individuals and groups seeking to advance alternative approaches to the management of labour, film had lasting significance as a medium of communication. Several dozen films were made in Europe and the United States between the 1950s and the 1970s, including by the leadership of the Natural Childbirth Trust, with the express purpose of promoting non-pharmacological methods of labour pain management. These ranged from ‘home videos’ to low-budget *cinéma-verité* documentaries to feature-length cinematic releases.^105^ As Dick-Read, Lamaze and their followers articulated the principles of ‘natural childbirth’ and ‘l'accouchement sans douleur’, they also developed the childbirth film as an enduring cultural form.

More generally, the history of *Childbirth without Fear* compels us to reflect further on the key roles the communications media played in reconstructing the meaning of birth in the mid-twentieth century.^106^ With the advent of the National Health Service and as hospital-based maternity care was made the norm during the post-war baby boom, discussion of childbirth became ever more open and less taboo. Film and television, along with print media, helped advocates of natural childbirth find a public for whom motherhood and family had become emblems of stability during the uncertainty of the Cold War. Conversely, this episode also invites consideration of the significance of childbirth in the histories of medical film and television, at crucial points in the development of these media. Along with other films depicting the moment of birth, *Childbirth without Fear* both pushed the boundaries and set the parameters for representing and viewing the facts of life on screen.

## Footnotes

1 Shirley Prendergast and Alan Prout, ‘The natural and the personal: reflections on birth films in schools’, British Journal of Sociology of Education (1985) 6, pp. 173–183; Lori Shorr, ‘Performing birth: the construction of female bodies in instructional childbirth videos’, Velvet Light Trap (1992) 29, pp. 3–14; Rebecca Feasey, From Happy Homemaker to Desperate Housewives: Motherhood and Popular Television, London: Anthem Press, 2012, pp. 147–176.

2 Eric Schaefer, ‘Exploitation as education’, in Devin Orgeron, Marsha Orgeron and Dan Streible (eds.), Learning with the Lights Off: Educational Film in the United States, Oxford: Oxford University Press, 2012, pp. 316–337; Kirsten Ostherr, Medical Visions: Producing the Patient through Film, Television and Imaging Technologies, Oxford: Oxford University Press, 2013, pp. 113–151; Benjamin Strassfeld, ‘A difficult delivery: debating the function of the screen and educational cinema through *The Birth of a Baby* (1938)’, Velvet Light Trap (2013) 72, pp. 44–57.

3 Judith Leavitt, Brought to Bed: Childbearing in America, 1750 to 1950, Oxford: Oxford University Press, 1986; Salim Al-Gailani, ‘Hospital birth’, in Nick Hopwood, Rebecca Flemming and Lauren Kassell (eds.), *Reproduction: From Antiquity to the Present Day*, Cambridge: Cambridge University Press, forthcoming.

4 Ornella Moscucci, ‘Holistic obstetrics: the origins of “natural childbirth” in Britain’, Postgraduate Medical Journal (2003) 79, pp. 168–1731269792010.1136/pmj.79.929.168PMC1742649; Amanda-Jane Raphael, ‘Natural childbirth in twentieth-century England: a history of alternative approaches to birth, 1940s–1990s’, unpublished PhD dissertation, Queen Mary University of London, 2010, at http://qmro.qmul.ac.uk/jspui/handle/123456789/1601.

5 Paula Michaels, Lamaze: An International History, Oxford: Oxford University Press, 2014; Wendy Kline, ‘Communicating a new consciousness: countercultural print and the home birth movement in the 1970s’, Bulletin of the History of Medicine (2015) 89, pp. 527–5562652167110.1353/bhm.2015.0065; Nick Hopwood, Peter Murray Jones, Lauren Kassell and Jim Secord, ‘Introduction: communicating reproduction’, Bulletin of the History of Medicine (2015) 89, pp. 379–4042652166610.1353/bhm.2015.0064PMC4655582.

6 Paula Michaels, ‘The sounds and sights of natural childbirth: films and records in antenatal preparation classes, 1950s–1980s’, Social History of Medicine (2017)10.1093/shm/hkw119PMC591436429713123, advance access, doi: 10.1093/shm/hkw119; Judith Walzer Leavitt, Make Room for Daddy: The Journey from Waiting Room to Birthing Room, Chapel Hill: University of North Carolina Press, 2009, pp. 149–152.

7 On natural childbirth as ‘ideology’ see Tess Cosslett, Women Writing Childbirth: Modern Discourses of Motherhood, Manchester: Manchester University Press, 1994, pp. 9–46; Moscucci, op. cit. (4).

8 Michaels, op. cit. (6).

9 On educational film see Orgeron, Orgeron and Streible, op. cit. (2). I have been able to reconstruct the history of *Childbirth without Fear* from Dick-Read's correspondence relating to the film, archived at the Wellcome Library, London (hereafter WL), files PP/GDR/C/18.1–4.

10 Grantly Dick-Read, Natural Childbirth, London: William Heinemann, 1933.

11 Grantly Dick-Read, Revelation of Childbirth: The Principles and Practice of Natural Childbirth, London: William Heinemann, 1942; Dick-Read, Childbirth without Fear, London: William Heinemann, 1944.

12 Moscucci, op. cit. (4).

13 William Nixon, ‘Childbirth and motherhood’, British Medical Journal (1950) 2, p. 1101.

14 Laura King, Family Men: Fatherhood and Masculinity in Britain, c.1914–1960, Oxford: Oxford University Press, 2015.

15 For entry points into the literature on gender and the family in the postwar years see Pat Thane, ‘Family life and “normality” in postwar British culture’, in Richard Bessell and Dirk Schimann (eds.), Life after Death: Approaches to a Cultural and Social History of Europe during the 1940s and 1950s, Cambridge: Cambridge University Press, 2003, pp. 193–210; Joanna Regulska and Bonnie Smith, Women and Gender in Postwar Europe: From Cold War to European Union, London: Routledge, 2012; Claire Langhamer, The English in Love: The Intimate Story of an Emotional Revolution, Oxford: Oxford University Press, 2013, p. 10. For discussions of how these values shaped attitudes to childbirth see Wendy Webster, Imagining Home: Gender, Race and National Identity 1945–1964, London: Routledge, 2005, pp. 92–94; Leavitt, op. cit. (6), pp. 86–119; Rebecca Jo Plant, Mom: The Transformation of Motherhood in Modern America, Chicago: The University of Chicago Press, 2010; Angela Davis, Modern Motherhood: Women and Family in England, 1945–2000, Manchester: Manchester University Press, 2012; Michaels, op. cit. (5), pp. 22–26.

16 Thomas Jeffcoate, ‘Childbirth without fear’, British Medical Journal (1954) 2, p. 1532.

17 Kelly Loughlin, ‘Spectacle and secrecy: press coverage of conjoined twins in 1950s Britain’, Medical History (2005) 49, pp. 197–2121589575610.1017/s0025727300008577PMC1088219; Ayesha Nathoo, Hearts Exposed: Transplants and the Media in 1960s Britain, Basingstoke: Palgrave Macmillan, 2009, pp. 33–56.

18 A. Noyes Thomas, Doctor Courageous: The Story of Dr. Grantly Dick Read, London: William Heinemann, 1957, p. 87.

19 Paula Michaels, ‘Comrades in the labor room: the Lamaze method of childbirth preparation and France's Cold War home front, 1951–1957’, American Historical Review (2010) 115, pp. 1031–1060; Michaels, op. cit. (5), pp. 45–68.

20 Lecture to Die Niederrheinisch-Westfälische Gesellschaft für Gynäkologie und Geburtshilfe, 5 October 1957, WL PP/GDR/C/88.

21 Michaels, op. cit. (5), pp. 62–92.

22 Translation of article on Lamaze in the French communist-backed journal *Les lettres françaises* (1953) by Frank Bamping, WL GC/106/1.

23 Dick-Read to J.E. Maltby (G-B Equipments), 16 October 1957, WL PP/GDR/C/18.3.

24 Thomas, op. cit. (18).

25 On Rank see Geoffrey Macnab, J. Arthur Rank and the British Film Industry, London: Routledge, 1993.

26 Dick-Read to J. Arthur Rank, 22 November 1946; L.W. Oliver (Technicolor Ltd) to Dick-Read, 21 May 1946; and Eddie Albert to Dick-Read, 25 August 1948, WL PP/GDR/C/18.1.

27 Brian Stanford, ‘The evolution of the medical film in Britain’, Canadian Medical Association Journal (1947) 57, pp. 385–387PMC159059020264812; Timothy Boon, ‘Medical film and television: an alternative path to the cultures of biomedicine’, in Mark Jackson (ed.), The Oxford Handbook of the History of Medicine, Oxford: Oxford University Press, 2011, pp. 617–634.

28 Michael Clark, ‘Audio-visual training materials in medicine’, in Chris Dry (ed.), Film and Television in Education: The Handbook of the British Universities Film and Video Council, 2nd edn, London: Chapman and Hall, 1995, pp. 21–29, 23.

29 Grantly Dick-Read, No Time for Fear, London: William Heinemann, 1955, p. xv.

30 Dick-Read to D.S. Garthorne, 5 May 1954, WL PP/GDR/C/18.1.

31 Scientific Film Association, Catalogue of Medical Films, rev. edn, London: Harvey and Blythe, 1952, pp. 55–63, 76–83.

32 ‘Obstetrics and gynaecology on the screen’, The Lancet (1949) 253, pp. 1107–1108.

33 ‘The making of a good medical film’, The Lancet (1951) 257, pp. 517–518.

34 Dick-Read to J. Arthur Rank, 1 March 1954, WL PP/GDR/C/18.1.

35 ‘The birth of a baby’, The Lancet (1940) 235, p. 325, and (1947) 249, p. 877; Nicholas Hallam, ‘The censor seldom wins’, *Daily Express*, 9 September 1947, p. 2. On *Birth of a Baby* and its reception in the United States see Strassfeld, op. cit. (2); Schaefer, op. cit. (2), p. 188.

36 Quotation from letter in British Board of Film Censors (BBFC) file on the 1949 Danish film *We Want a Baby*, released in Britain in 1953, BBFC archive, London.

37 Maternity in Great Britain: A Survey of Social and Economic Aspects of Pregnancy and Childbirth Undertaken by a Joint Committee of the Royal College of Obstetrics and Gynaecology and the Population Investigation Committee, Oxford: Oxford University Press, 1948, pp. 22–23.

38 William Nixon, ‘Antenatal care’, Health Education Journal (1948) 6, pp. 71–741891706010.1177/001789694800600207; ‘Where mothers learn’, Health Education Journal (1957) 15, pp. 216–222.

39 Timothy Boon, ‘Health education films in Britain, 1919–1939: productions, genres and audiences’, in Graeme Harper and Andrew Moor (eds.), Signs of Life: Cinema and Medicine, London: Wallflower, 2005, pp. 45–57.

40 John Burton, ‘The film and public health’, Health Education Journal (1953) 11, pp. 182–187.

41 Burton, op. cit. (40), p. 183.

42 Andreas Killen, ‘Weimar cinema between Enlightenment and hypnosis’, in Michael Laffan and Max Weiss (eds.), Facing Fear: The History of an Emotion in Global Perspective, Princeton, NJ: Princeton University Press, 2012, pp. 91–113.

43 Dick-Read, op. cit. (11), p. 69.

44 William Hickey, ‘Close-up of birth’, *Daily Express*, 9 February 1940, p. 4; Max Caulfield, Mary Whitehouse, London: Mowbrays, 1975, p. 19; Barbara Crowther, ‘The partial picture: framing the discourse of sex in the British educative films of the early 1930s’, in Lutz Sauerteig and Roger Davidson (eds.), Shaping Sexual Knowledge: A Cultural History of Sex Education in Twentieth-Century Europe, London: Routledge, 2009, pp. 176–196.

45 Editorial, *Science and Film* (1953) 1, pp. 1–2; Burton, op. cit. (40); Scott Curtis, ‘Dissecting the medical training film’, in Marta Braun, Charles Keil, Rob King, Paul Moore and Louis Pelletier (eds.), Beyond the Screen: Institutions, Networks and Publics of Early Cinema, New Barnet: John Libbey, 2012, pp. 161–167.

46 Dick-Read, op. cit. (11), p. 69; correspondence between Dick-Read and American filmmaker Leslie Shepard, February–March 1956, WL PP/GDR/C/24. For a similar view see ‘Problems of maternal mortality’, *The Guardian*, 29 April 1937, p. 14.

47 Grantly Dick-Read, Childbirth without Fear, New York: Harper and Row, 1953, pp. ix–x.

48 For a similar view see ‘Editorial: moving pictures’, Health Education Journal (1955) 13, p. 68.

49 Thomas, op. cit. (18), p. 208.

50 Dick-Read to J. Arthur Rank, 1 July 1954, WL PP/GDR/C/18.1.

51 Dick-Read to R.E. King (G-B Equipments, Ltd), 4 September 1954, WL PP/GDR/C/18.1.

52 Dick-Read, op. cit. (29).

53 Dick-Read to R.E. King, 9 November 1954, WL PP/GDR/C/18.1.

54 As Michaels, op. cit. (5), p. 21, has noted, didactic materials promoting the Read method positioned the mother as openly deferential to male medical authority, even as they touted women's agency.

55 A copy of the film is held at the British Film Institute archive (identifier no 15912).

56 ‘Why was this sex film cut?’, *Daily Express*, 24 May 1950, 7.

57 R.E. King to Dick-Read, 6 September 1954, WL PP/GDR/C/18.1.

58 R.E. King to Dick-Read, 16 November 1954, WL PP/GDR/C/18.1.

59 Dick-Read to J.E. Maltby, 20 August 1956, WL PP/GDR/C/18.3.

60 Thomas, op. cit. (18).

61 On the significance of language and mode of address see Anne Karpf, ‘Constructing and addressing the “ordinary devoted mother”’, History Workshop Journal (2014) 78, pp. 82–106.

62 ‘Editorial: moving pictures’, Health Education Journal (1955) 13, pp. 68–69; Burton, op. cit. (40), p. 182. See also Mary Field, ‘The film in education’, Health Education Journal (1944) 2, pp. 172–175.

63 Dick-Read to Otto Busse, 13 September 1957, WL PP/GDR/C/88.

64 Introductory text for *Childbirth without Fear*, Dick-Read to R.E. King, 2 August 1955, WL PP/GDR/C/18.2.

65 Dick-Read, ‘Physiological childbirth’, WL PP/GDR/C/18.1.

66 ‘Childbirth without Fear – notes’, WL PP/GDR/C/18.1.

67 Press cuttings of reviews of screenings from *Nursery World*, 21 April 1955; *Nursing Mirror*, 22 April 1955; *Nursing Times*, 22 April 1955; *Parents*, 22 June 1955, all in WL PP/GDR/C/133.

68 Michal Shapira, The War Inside: From Collective to Domestic Citizenship, Cambridge: Cambridge University Press, 2013, pp. 112–137; Karpf, op. cit. (61). On Cold War ‘mother love’ more generally see Plant, op. cit. (15); Marga Vicedo, ‘Cold War emotions: mother love and the war over human nature’, in Mark Solovey and Hamilton Cravens (eds.), Cold War Social Science, Basingstoke: Palgrave Macmillan, 2012, pp. 233–249.

69 Dick-Read, op. cit. (65).

70 Dick-Read to J.E. Maltby, 20 August 1956, WL PP/GDR/C/18.3.

71 J.E. Maltby to GDR, 4 April 1956, WL PP/GDR/C/18.3.

72 Reviews in *The Lancet* (1955) 265, p. 1231; *Medical Press*, 4 May 1955, cutting in WL PP/GDR/C/133.

73 *The Guardian*, 10 February 1956, p. 7; *Daily Sketch*, 10 February 1956, cutting in WL PP/GDR/C/133.

74 *Parents*, 22 June 1955, original emphasis, cutting in WL PP/GDR/C/133.

75 ‘Study of natural childbirth’, *The Times*, 15 May 1956, p. 6.

76 *Nursery World*, 20 June 1957, cutting in WL PP/GDR/C/134.

77 Clifford Davis, ‘Birth of a baby – on TV’, *Daily Mirror*, 4 February 1957, cutting in WL PP/GDR/C/133.

78 Joy Leman, ‘Programmes for women in 1950s British television’, in Helen Baehr and Gillian Dyer (eds.) Boxed In: Women and Television, London: Pandora, 1987, pp. 73–95; Janet Thumim (ed.), Inventing Television Culture: Men, Women and the Box, Oxford: Oxford University Press, 2004.

79 Mary Irwin, ‘What women want on television: Doreen Stephens and BBC television programmes for women, 1953–1964’, Westminster Papers (2011) 8, pp. 99–122.

80 ‘TV cameras in mothers’ ward’, *Daily Express*, 15 November 1956, p. 3.

81 Victoria Wegg-Prosser, ‘*This Week* in 1956: the introduction of current affairs on ITV’, in Thumim, op. cit. (78), pp. 195–206, 198; Ien Ang, Desperately Seeking the Audience, London: Routledge, 1991; Su Holmes, Entertaining Television: The BBC and Popular Television in the 1950s, Manchester: Manchester University Press, 2008.

82 *Daily Mirror*, 5 February 1957, cutting in WL PP/GDR/C/133.

83 On Dimbleby and *Panorama* see Asa Briggs, The History of Broadcasting in the United Kingdom: Competition, vol. 5, Oxford: Oxford University Press, 1995, pp. 164–165. The critics were Anne Scott-James, woman's editor at the *Sunday Express*, and an obstetrician who, under strict professional conventions regarding doctor anonymity, remained unnamed.

84 *Manchester Daily Express*, 5 February 1957, cutting in WL PP/GDR/C/133.

85 *Daily Sketch*, 10 February 1956; *Daily Sketch*, 5 February 1957, cuttings in WL PP/GDR/C/133. On earlier attempts by the *Sketch* to orchestrate a moral backlash against sexual content in rival publications see Adrian Bingham, ‘The British popular press and venereal disease during the Second World War’, Historical Journal (2005) 48, pp. 1055–1076, 1063.

86 For instance, *Daily Mirror*, 5 February 1957, cutting in WL PP/GDR/C/133.

87 *News Chronicle*, 5 February 1957, cutting in WL PP/GDR/C/133.

88 ‘Birth film was sincerely presented’, *Edinburgh Evening Dispatch*, 18 February 1957, cutting in WL PP/GDR/C/134.

89 *Manchester Daily Express*, 5 February 1957; *Birmingham Evening Dispatch*, 5 February 1957, cuttings in WL PP/GDR/C/133.

90 Maurice Richardson, ‘Cupid and Charon’, *The Observer*, 10 February 1957, p. 11.

91 Mass-Observation film reports January 1937–December 1948, 17-4-F, Mass-Observation Online Archive.

92 Kate Fisher, Birth Control, Sex, and Marriage in Britain, 1918–60, Oxford: Oxford University Press, 2006, p. 240; Adrian Bingham, Family Newspapers: Sex, Private Life and the British Popular Press, 1918–1978, Oxford: Oxford University Press, 2009; Bingham, ‘The “K-Bomb”: social surveys, the popular press, and British sexual culture in the 1940s and 1950s’, Journal of British Studies (2011) 50, pp. 156–179.

93 *Exeter Express and Echo*, 8 February 1957, cutting in WL PP/GDR/C/133.

94 ‘Father sees baby born on TV’, *Daily Express*, 17 July 1957, p. 7. BBC television, *Your Life in Their Hands: Caesarean Section*, 20 March 1963; *Having Your Baby*, ten-part series on maternity and baby care starting 11 October 1964.

95 BBFC file on *We Want a Baby*, letter to London and Overseas Film Services, 15 September 1958.

96 Transcript of BBC *Panorama*, ‘Natural childbirth’, 4 February 1957, in WL PP/GDR/C/18.3.

97 *Parents*, May 1957, pp. 20–22, March 1958, p. 39.

98 *Parents*, May 1957, p. 22.

99 Ann Friedrich, ‘I photographed my baby's birth’, *Parents*, January 1958, pp. 38–42.

100 Jenny Kitzinger, ‘Strategies of the early childbirth movement: a case-study of the Natural Childbirth Trust’, in Jo Garcia, Robert Kilpatrick and Martin Richards (eds.), The Politics of Maternity Care: Services for Childbearing Women in Twentieth-Century Britain, Oxford: Clarendon Press, 1990, pp. 92–115.

101 Robert G. Moeller, Protecting Motherhood: Women and the Family in the Politics of Postwar West Germany, Berkeley: University of California Press, 1993.

102 Dick-Read's correspondence with Dieter Fritko, February 1957–February 1958, WL PP/GDR/C/23.

103 Michaels, op. cit. (5), pp. 75–76; Kitzinger, op. cit. (100); Raphael, op. cit. (4).

104 Linton Snaith and Alan Coxon, Dick-Read's Childbirth without Fear: The Principles and Practice of Natural Childbirth, London: William Heinemann, 1968, p. 233.

105 Suggestive lists in moving-image collection of Lamaze International, 1956–2010, MP-47.1-MP-47.13; Vt-112.1-Vt-112.8, Arthur and Elizabeth Schlesinger Library, Harvard University; and Flora Hommel Papers, Walter P. Reuther Library, Wayne State University, Detroit.

106 Kitzinger, op. cit. (100).

